# Selective vulnerability of the cerebral vasculature to blast injury in a rat model of mild traumatic brain injury

**DOI:** 10.1186/2051-5960-2-67

**Published:** 2014-06-17

**Authors:** Miguel A Gama Sosa, Rita De Gasperi, Pierce L Janssen, Frank J Yuk, Pamela C Anazodo, Paul E Pricop, Alejandro J Paulino, Bridget Wicinski, Michael C Shaughness, Eric Maudlin-Jeronimo, Aaron A Hall, Dara L Dickstein, Richard M McCarron, Mikulas Chavko, Patrick R Hof, Stephen T Ahlers, Gregory A Elder

**Affiliations:** General Medical Research Service, James J. Peters Department of Veterans Affairs Medical Center, 130 West Kingsbridge Road, Bronx, New York USA; Research and Development Service, James J. Peters Department of Veterans Affairs Medical Center, Bronx, New York USA; Neurology Service, James J. Peters Department of Veterans Affairs Medical Center, Bronx, New York USA; Department of Psychiatry, Icahn School of Medicine at Mount Sinai, New York, New York, USA; Fishberg Department of Neuroscience, Icahn School of Medicine at Mount Sinai, New York, New York, USA; Department of Geriatrics and Palliative Care, Icahn School of Medicine at Mount Sinai, New York, New York, USA; Department of Neurology, Icahn School of Medicine at Mount Sinai, New York, New York, USA; Friedman Brain Institute, Icahn School of Medicine at Mount Sinai, New York, New York, USA; Operational and Undersea Medicine Directorate, Naval Medical Research Center, Silver Spring, Maryland USA

**Keywords:** Blast, Rat, Traumatic brain injury, Vascular pathology

## Abstract

**Background:**

Blast-related traumatic brain injury (TBI) is a common cause of injury in the military operations in Iraq and Afghanistan. How the primary blast wave affects the brain is not well understood. The aim of the present study was to examine whether blast exposure affects the cerebral vasculature in a rodent model. We analyzed the brains of rats exposed to single or multiple (three) 74.5 kPa blast exposures, conditions that mimic a mild TBI. Rats were sacrificed 24 hours or between 6 and 10 months after exposure. Blast-induced cerebral vascular pathology was examined by a combination of light microscopy, immunohistochemistry, and electron microscopy.

**Results:**

We describe a selective vascular pathology that is present acutely at 24 hours after injury. The vascular pathology is found at the margins of focal shear-related injuries that, as we previously showed, typically follow the patterns of penetrating cortical vessels. However, changes in the microvasculature extend beyond the margins of such lesions. Electron microscopy revealed that microvascular pathology is found in regions of the brain with an otherwise normal neuropil. This initial injury leads to chronic changes in the microvasculature that are still evident many months after the initial blast exposure.

**Conclusions:**

These studies suggest that vascular pathology may be a central mechanism in the induction of chronic blast-related injury.

## Introduction

Blast exposure is a rare cause of traumatic brain injury (TBI) in civilian life [1]. However, blast injury has longstanding importance in military head trauma [2] and recently, there has been renewed interest in blast-related TBI because of the frequency of this injury in the conflicts in Iraq and Afghanistan [3]. Indeed, it has been estimated that 10-20% of returning veterans from these conflicts have suffered a TBI with blast exposure from improvised explosive devices (IED) being the most common cause [3, 4].

Whereas in the military operations in Iraq and Afghanistan most attention initially focused on the moderate-to-severe end of the TBI spectrum, it soon became apparent that mild TBIs (mTBI) were much more common and frequently not recognized at the time of the initial injury [3]. Single or multiple blast-related mTBIs have been associated with chronic neurological and psychiatric symptoms [3]. There have also been concerns that blast injury, like forms of non-blast TBI (nbTBI), may be associated with the later development of progressive neurodegenerative disorders such as Alzheimer’s disease or chronic traumatic encephalopathy (CTE) [5] and indeed multiple cases of CTE have been described in veterans from Iraq and Afghanistan [6, 7].

How the primary blast wave itself affects the brain is not well-understood [8]. In particular, whether blast TBI activates primary and secondary injury cascades similar to those activated in nbTBI is unknown. We previously reported the development of a rat model of blast-induced mTBI. In this model, exposures up to 74.5 kPa, while representing a blast level that is transmitted to the brain [9], do not result in persistent neurological impairments or lung damage [10], although the animals exhibit a variety of chronic behavioral traits that mimic those found in posttraumatic stress disorder [11].

In a previous study of rats subjected to 74.5 kPa blast exposures, we also described a type of shear injury in the brain that has not been described in nbTBI models and appears to be unique to blast-associated brain injury [12]. In the present study we explored the effects of blast exposure on the cerebral vasculature. We describe a selective vascular pathology that is visible acutely. This pathology extends beyond the margins of shear-related injuries and leads to chronic changes in the microvasculature that are evident many months after the initial injury. These studies suggest that vascular pathology may be a central mechanism in the induction of chronic blast-related injury.

## Materials and methods

### Blast overpressure exposure

All studies were approved by the Institutional Animal Care and Use Committees of the Naval Medical Research Center (Silver Spring, MD, USA), and the James J. Peters VA Medical Center (Bronx, NY, USA). Studies used adult male Long Evans hooded rats (2 months-old, 250-350 g; Charles River Laboratories International, Wilmington, MA, USA). Animals were individually housed at a constant 22°C on a 12:12 hour light cycle in standard clear plastic cages equipped with laboratory animal bedding and nesting paper. Access to food and water was *ad libitum*.

Rats were subjected to blast overpressure exposures of compressed air using the Walter Reed Army Institute of Research (WRAIR) shock tube as previously described [10, 12]. Blast exposures occurred under isoflurane anesthesia. Rats were randomly assigned to sham or blast conditions with the head facing the blast overpressure without any body shielding resulting in a full body exposure to the blast wave. Further details of the physical characteristics of the blast wave have been described elsewhere [10, 11]. Blast-exposed animals received one or three 74.5 kPa exposures. Animals that received three exposures received one exposure per day for three consecutive days. Except for blast overpressure exposures, control animals were treated identically including receiving anesthesia and being placed in the blast tube. Animals were transferred to the James J. Peters VA Medical Center within 10 days of blast exposure. The number of animals per condition examined in this study is indicated in Table 1.

**Table 1:** **Experimental groups**

Blast condition	Time harvested post-blast exposure	N blast exposed	N control
1 × 74.5 kPa	24 hours	7	6
3 × 74.5 kPa	24 hours	7	6
3 × 74.5 kPa	6-10 months	23	15

### Histopathological and immunohistochemical analysis

Animals were anesthetized with ketamine/xylazine/acepromazine (65:13:2 mg/kg) and transcardially perfused with cold 4% paraformaldehyde in phosphate-buffered saline (PBS). Hematoxylin-eosin (H&E) staining and immunohistochemical analyses were performed on 50 μm-thick coronal Vibratome sections as previously described [13]. The primary antibodies used were a rabbit polyclonal anti-collagen IV antiserum (1:500; Abcam, Cambridge, MA, USA, ab6586), a rabbit polyclonal anti-laminin (1:150; Sigma-Aldrich, St. Louis, MO, USA, L9393), a mouse monoclonal anti-α-smooth muscle actin (α-SMA, 1:500; Sigma, A5228), and a rat monoclonal anti-glial fibrillary acidic protein (GFAP, 1:500, gift of Dr. Virginia Lee, University of Pennsylvania, Philadelphia PA, USA). Sections were blocked with Tris-buffered saline (TBS; 50 mM Tris–HCl, 0.15 M NaCl, pH 7.6), containing 0.1% Triton X-100/5% goat serum (TBS-TGS) for 1 hour, and incubated overnight with the primary antibodies in TBS-TGS at room temperature. After several washes with PBS for 1 hour, immunostaining was detected with species-specific AlexaFluor 488- and 568-conjugated secondary antibodies (1:300; Molecular Probes, Burlingame CA, USA) for 2 hours in TBS-TGS. Nuclei were counterstained with 1 μg/ml 4′,6-diamidino-2-phenylindole (DAPI). For some studies immunostaining was performed on pepsin-digested sections as previously described [14]. For pepsin pretreatment, sections were treated with 1 mg/ml pepsin (Dako, Carpinteria, CA, USA) in 3% acetic acid for 50 minutes at 37°C, extensively washed with PBS, blocked and immunostained. Apoptotic cells were identified by TUNEL analyses using a commercial kit that incorporates fluorescein 2´-deoxyuridine, 5´-triphosphate (dUTP) to free 3’-OH termini at DNA strand breaks in the presence of terminal transferase (Roche, Indianapolis, IN, USA). Sections were permeabilized in 0.1% Triton X-100 in 50 mM Tris–HCl, 0.15 M NaCl, pH 7.6 for 1 hour, extensively washed with TBS, TUNEL stained, blocked and immunostained for α-SMA. Stained sections were photographed on a Zeiss AxioImager microscope using the AxioVision Release 4.3 software program (Zeiss, Thornwood, NY, USA), a Nikon Eclipse E400 connected to a DXC-390 CCD camera (Nikon, Melville, NY, USA) or a Zeiss LSM 710 confocal microscope. Digital images were color balanced using Adobe Photoshop 11.0 (Adobe Systems, San Jose, CA, USA).

### Electron microscopy

Electron microscopy (EM) was performed using protocols optimized to study the ultrastructure of blood vessels as previously described [15, 16]. Rats were anesthetized and perfused as described above with 2% paraformaldehyde containing 2% glutaraldehyde in 0.1 M sodium phosphate buffer, pH 7.0. Tissue was removed and postfixed in the same fixative overnight. Fixed brains were placed on a rat brain slicer matrix and coronal slices containing the frontal cortex were excised and processed for EM. Freeze substitution and low-temperature embedding of the specimens was performed as described elsewhere [16–18]. Slices were cryoprotected by immersion in 4% D-glucose, followed by increasing concentrations of glycerol (from 10% to 30% in phosphate buffer; v/v) and plunged rapidly into liquid propane cooled by liquid nitrogen (-190°C) in a Universal Cryofixation System KF80 (Reichert-Jung, Vienna, Austria). The samples were immersed in 0.5% uranyl acetate in anhydrous methanol (-90°C, 24 hours) in a cryosubstitution AFS unit (Leica, Vienna, Austria). The temperature was raised from -90°C to -45°C in steps of 4°C/hour. After washing with anhydrous methanol, the samples were infiltrated with Lowicryl HM20 resin (Electron Microscopy Sciences, Fort Washington, PA, USA) at -45°C. Polymerization with ultraviolet light (360 nm) was performed for 48 hours at -45°C, followed by 24 hours at 0°C. Ultrathin sections (70 nm) were cut with a diamond knife on a Reichert-Jung ultramicrotome and mounted on nickel grids using a Coat-Quick adhesive pen (Electron Microscopy Sciences). Sections were imaged on a Hitachi 7700 electron microscope (Tokyo, Japan) and photographed with an Advantage CCD camera (Advanced Microscopy Techniques, Danvers, MA, USA). Images were adjusted for brightness and contrast using Adobe Photoshop 11.0.

## Results

We examined rats that had been subjected to single or multiple (three) 74.5 kPa blast exposures that were sacrificed 24 hours or from 6–10 months after the last blast exposure. Table 1 contains a summary of the animals examined which include some that were used in a previous study [12]. There was no mortality in any of the blast-exposed or control groups. In a prior study, we reported that while causing no generalized histopathology, such exposures could cause hemorrhage in the choroid plexus and lateral ventricles as well as induce shear-related lesions, which may be unique to blast-induced brain injury [12]. These focal lesions frequently appeared to follow the course of penetrating cortical vessels and microhemorrhages were sometimes observed with these lesions.

**Figure 1:**
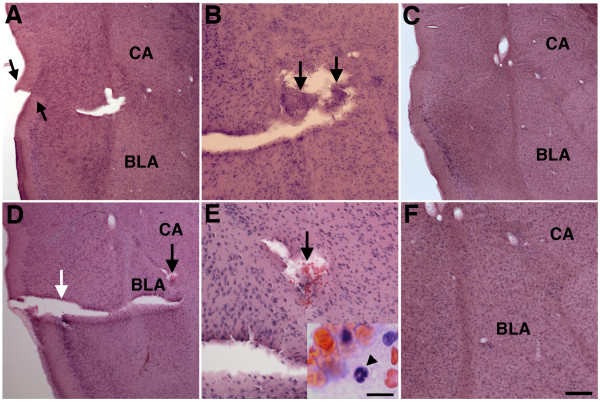
**Blast-induced shear-related injuries in brain.** H&E stained sections from a rat sacrificed 10 months after receiving 3 × 74.5 kPa blast exposures. Panels **A**, **B** and **D** are from serial sections taken 500 μm apart. Panel **A** shows a disruption of the normal continuity of the superficial cortical layers of the agranular insular cortex (arrows). The basolateral (BLA) and central amygdaloid (CA) nuclei are indicated. In panel **B**, a lesion contains tissue (arrows) that appears to have been avulsed and displaced. Panels **D** and **E** show two lesions. One appears to follow the course of a branch of the corticoamydaloid artery (white arrow). There is also an area of hemorrhage (black arrow), which is shown at higher power in panel **E**. The inset in panel **E** shows a higher power image of the hemorrhage itself. A polymorphonuclear leukocyte is indicated by an arrowhead. Sections from control animals are shown in panels **C** and **F**. Scale bar: 200 μm **A**, **C** and **D**; 100 μm **B** and **F**; 50 μm **E**. Scale bar for inset in panel **E**: 10 μm.

**Figure 2:**
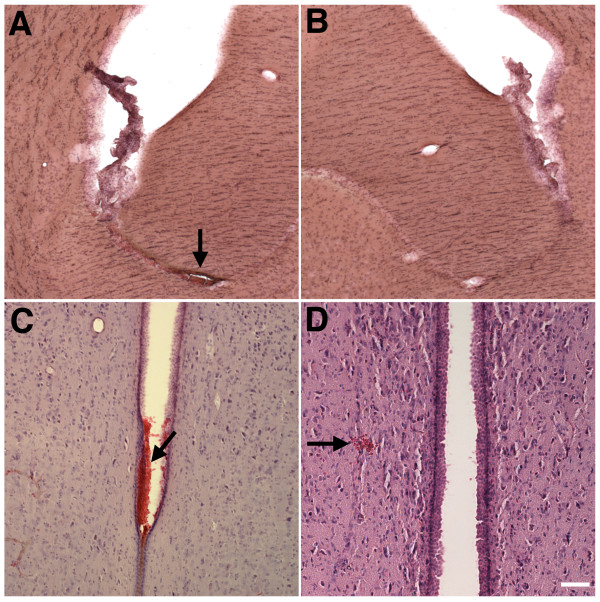
**Blast-induced intraventricular and intracerebral hemorrhage 10 months after blast exposure.** H&E stained sections from rats sacrificed 10 months after receiving 3 × 74.5 kPa blast exposures. Panel **A** shows a ventricular hemorrhage (arrow) near the fimbria that may have originated in the choroid plexus. Panel **B** shows a matching section from a control animal. Panels **C** and **D** show hemorrhages (arrows) in the third ventricle **(C)** and next to the periventricular nucleus **(D)** from two additional blast-exposed rats. Scale bar: 200 μm **A**-**B**; 100 μm **C**-**D**.

Examples of such lesions are illustrated in Figure 1 which shows H&E-stained sections from a rat that was exposed to single 74.5 kPa exposures delivered daily for three consecutive days (3 × 74.5 kPa) and sacrificed 10 months after the last exposure. The lesion in Figure 1 follows the course of a branch of the corticoamygdaloid artery and extends through the agranular insular cortex, external capsule and basolateral amygdala. The lesion produced a misalignment of the superficial cortical layers of the agranular insular cortex (Figure 1A, arrows). A cavity deep to the superficial lesion contains tissue that was likely displaced by the blast (Figure 1B, arrows). Interestingly, despite the fact that this animal had received its last blast exposure 10 months previously, a focal hemorrhage extending into the central amygdaloid nucleus was present (Figure 1D and E, black arrow). The presence of polymorphonuclear leukocytes within this hemorrhage (inset Figure 1E) indicates that it was of recent origin. Indeed intraventricular hemorrhage was observed in 4 out of 23 blast-exposed animals examined between 6 and 10 months post-exposure (Figure 2, A and C, arrows) and isolated intraparenchymal microhemorrhages could sometimes also be observed (Figure 2D, arrow). Shear-related lesions often leading to disrupted cortical organization and in some cases resulting in unusual tissue realignments were found in many cortical areas in rats exposed to single or 3 × 74.5 kPa blast exposures studied both acutely and chronically [12].

### Blast induces vascular pathology at the margins of focal lesions

**Figure 3:**
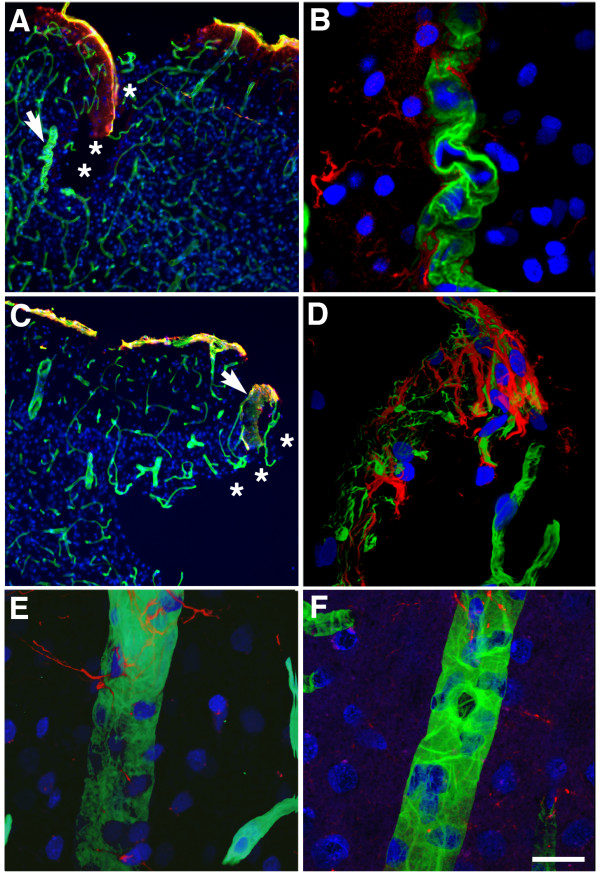
**Blast-induced vascular pathology at the margins of shear-related injuries.** Sections of the primary visual cortex from a rat that received 3 × 74.5 kPa blast exposures and was sacrificed 10 months after the last exposure. Sections were immunostained for collagen IV (green) and GFAP (red). Nuclei were stained with DAPI (blue) and the sections were imaged by confocal microscopy. Panel **B** shows a higher power image of the vessel indicated by the arrow in panel **A**. Panel **D** shows a higher power image of the region in panel **C** indicated by the arrow. The site of a cortical tear is indicated by asterisks in panels **A** and **C**. Note the tortuosity of the vessel (likely an arteriole) in panel **B**. In panel **D** collagenous remnants appear in the presence of gliosis. Panels **E** shows a normal appearing vessel from the same animal located away from the immediate borders of the lesions. Panel **F** shows a normal vessel from a control animal. Scale bar: 200 μm **A** and **C**; 20 μm **B**, **D**, **E** and **F**.

**Figure 4:**
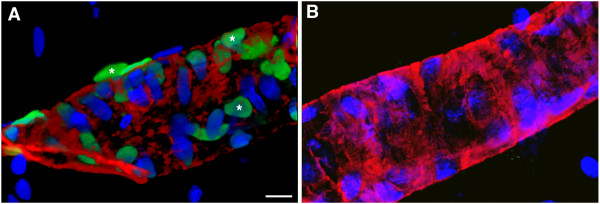
**Blast-induced vascular apoptosis at the margins of a shear-related injury.** Panel **A** shows images of a hippocampal vessel from the brain of a rat that received 3 × 74.5 kPa blast exposures and was sacrificed 24 hours after the last exposure. The section was labeled for TUNEL (green) and immunostained for α-smooth muscle actin (red). Nuclei were stained with DAPI (blue) and sections were imaged by confocal microscopy. Three TUNEL-labeled cells are indicated by asterisks. Panel **B** shows a vessel from a control animal. No TUNEL-labeled cells are apparent in the control. Scale bar: 10 μm.

Because lesions typically appeared to follow the patterns of penetrating cortical vessels, we explored whether the cerebral vasculature might be more sensitive to blast induced injury than other cerebral elements. Initially we examined the status of the vasculature near focal lesions and found that vascular pathology at the margins of lesions was common. Figure 3 shows collagen IV-immunostained sections of the primary visual cortex of a rat that received 3 × 74.5 kPa blast exposures and was sacrificed 10 months after the last exposure. Tortuous vessels (Figure 3A, arrow and B) and vascular collagenous remnants associated with GFAP-positive cells (astrogliosis) were observed near the margins of a lesion (Figure 3C, arrow and D). In animals examined 24 hours after blast exposure, apoptotic cells were seen in vessels near the margins of lesions. For example, Figure 4A shows the presence of apoptotic cells (representative TUNEL-positive cells are indicated by asterisks) in a major hippocampal vessel located at the margin of a lesion in the brain of a rat that had received 3 × 74.5 kPa blast exposures and was sacrificed 24 hours after the last exposure.

### Blast induces chronic vascular pathology that extends beyond the margins of focal lesions

To investigate whether blast exposure induces vascular pathology beyond the margins of focal lesions and to assess vascular integrity more generally, we performed immunohistochemical analysis of the vascular extracellular matrix components collagen IV and laminin. We and others have found that efficient immunostaining of collagen IV and laminin in the mature adult rodent brain requires a proteolytic epitope unmasking step [14, 19, 20]. In a wild-type mature adult brain, widespread immunostaining for collagen IV and laminin can be reliably achieved only following pepsin pretreatment [14]. However, under certain pathological conditions that include vascular degeneration, immunodetection of collagen IV and laminin occurs without antigen retrieval [13].

In all of the chronic blast-exposed animals examined (n = 3), immunostaining of vascular elements with laminin and collagen IV was observed without pepsin pretreatment in areas that extended beyond the edges of focal lesions. In contrast, in brain regions more distant from focal lesions, collagen IV and laminin were detected in the microvasculature only after pepsin pretreatment. In non-blast controls (n = 3), only patchy collagen IV and laminin immunostained regions were observed without pepsin pretreatment.

**Figure 5:**
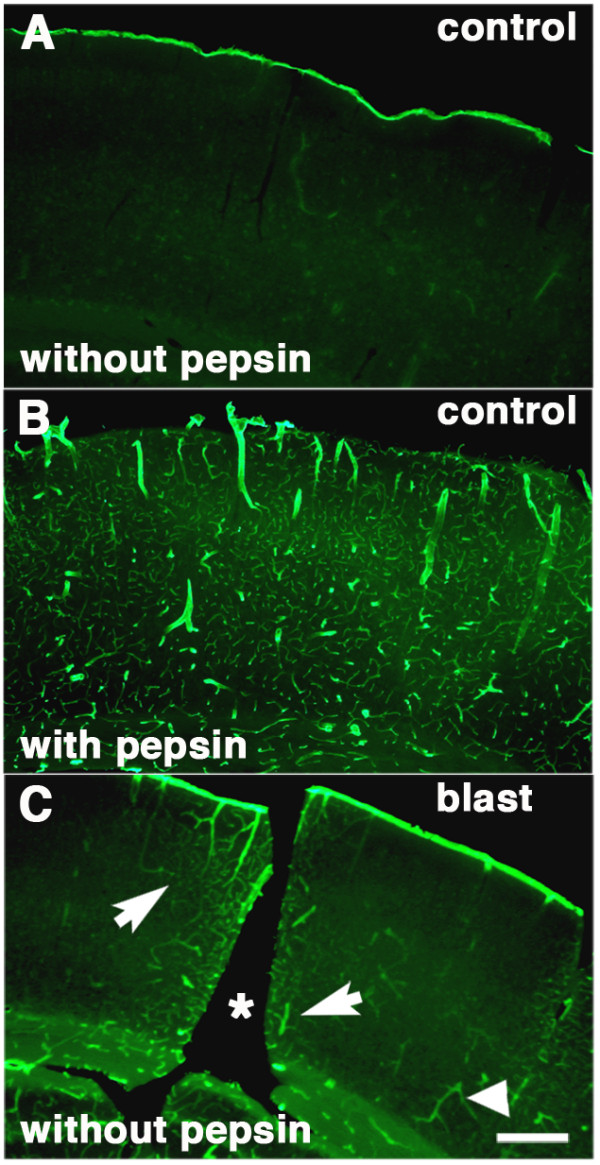
**Altered laminin in the microvascular extracellular matrix of blast-exposed animals.** Laminin immunostaining in the visual cortex of a control rat without **(A)** or with pepsin pretreatment **(B)**. Note the extensive laminin immunostaining after pepsin pre-treatment. Shown in panel **C** is a section of the visual cortex from a rat that received 3 × 74.5 kPa blast exposures and was sacrificed 10 months after the last blast exposure. The sections have been immunostained for laminin without pepsin pre-treatment. A focal lesion is visible (asterisk). Note the immunostained vessels both adjacent to (arrows) and distant from (arrowhead) the lesion. Scale bar: 400 μm.

Figure 5 shows the effect of pepsin pretreatment on laminin immunostaining in the cortex of a control brain. Without pepsin pretreatment (Figure 5A) no laminin immunostaining is observed, while after treatment (Figure 5B) extensive laminin immunostaining is present. Figure 5C shows the visual cortex from a rat that received 3 × 74.5 kPa blast exposures and was sacrificed 10 months after the last exposure. With pepsin pretreatment, laminin immunostaining in the blast exposed would be as apparent as in the pepsin-treated control (data not shown). However, as shown in Figure 5C even without pepsin pretreatment laminin immunostaining is visible around the focal lesion unlike the control shown in Figure 5A, where no laminin immunostaining is observed.

**Figure 6:**
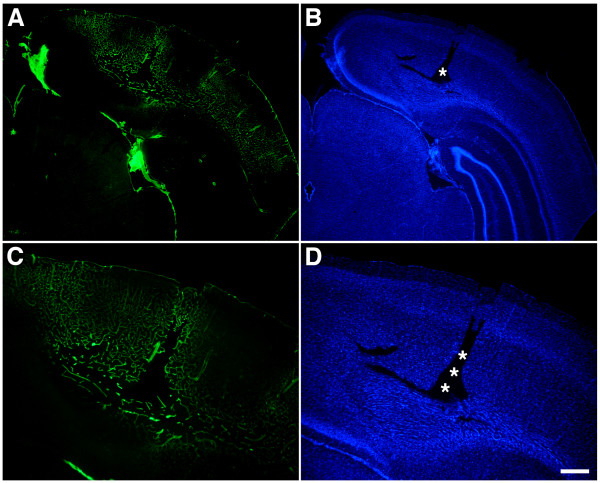
**Altered collagen IV immunostaining in the microvasculature of blast-exposed animals.** Different magnifications of a focal lesion in the visual cortex from a rat that received 3 × 74.5 kPa blast exposures and was euthanized 10 months after the last blast exposure. Sections were immunostained for collagen IV without pepsin pretreatment **(A, C)**. Nuclei were stained with DAPI **(B, D)**. Asterisks indicate the site of the focal cortical lesion. Note the extensive collagen IV immunostaining extending from the lesion. Scale bar: 750 μm **A**-**B**; 400 μm **C**-**D**.

**Figure 7:**
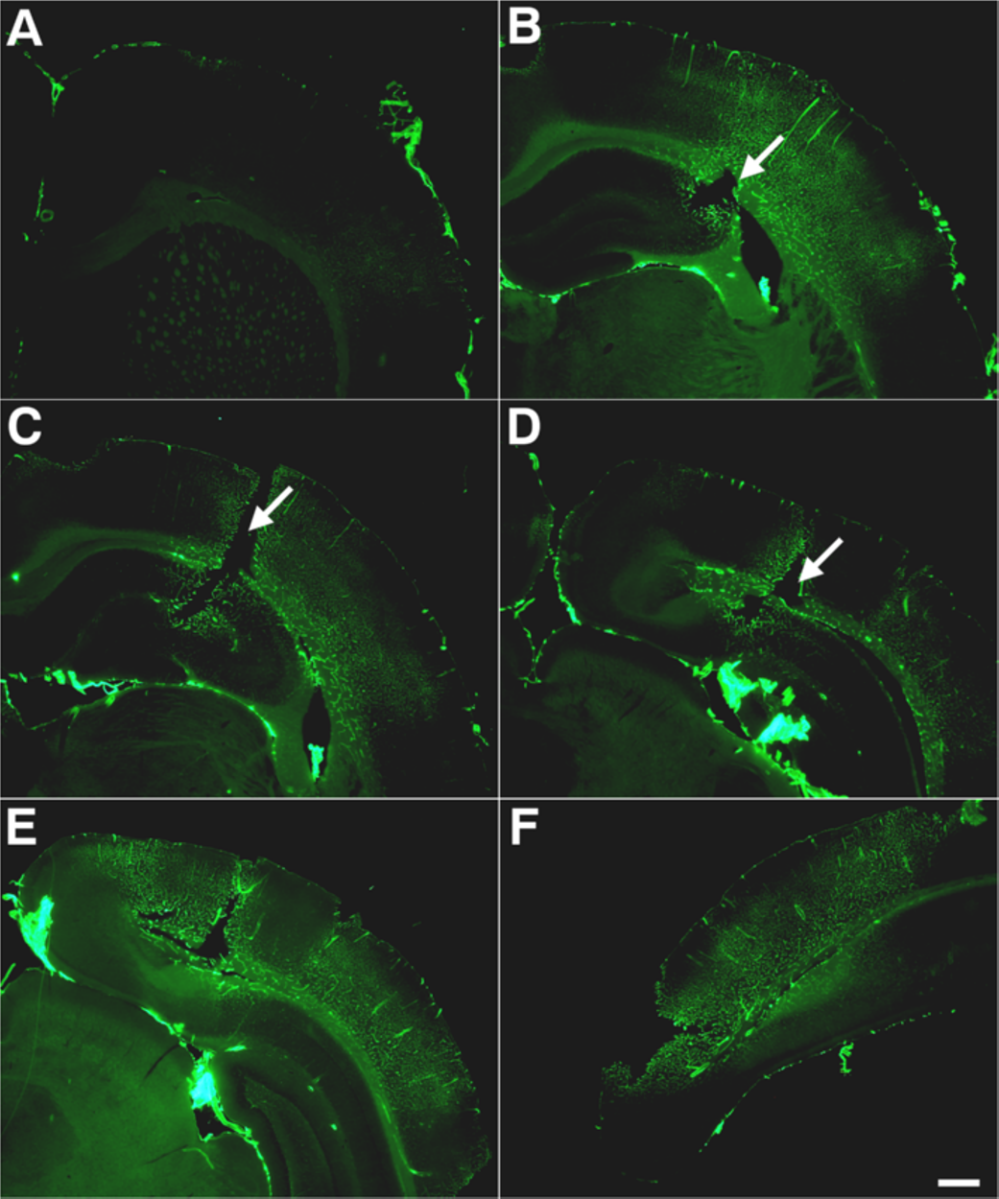
**Lateral and rostro-caudal extension of abnormal collagen IV immunostaining next to a blast-induced focal cortical lesion.** Collagen IV immunostaining of serial sections **(A-F)** taken 1200 μm apart around the focal cortical lesion illustrated in Figures 5 and 6. Lesion is indicated by arrows in panels **B**, **C** and **D**. Note the extensive lateral and rostro-caudal area of altered collagen IV immunostaining. Scale bar: 750 μm.

**Figure 8:**
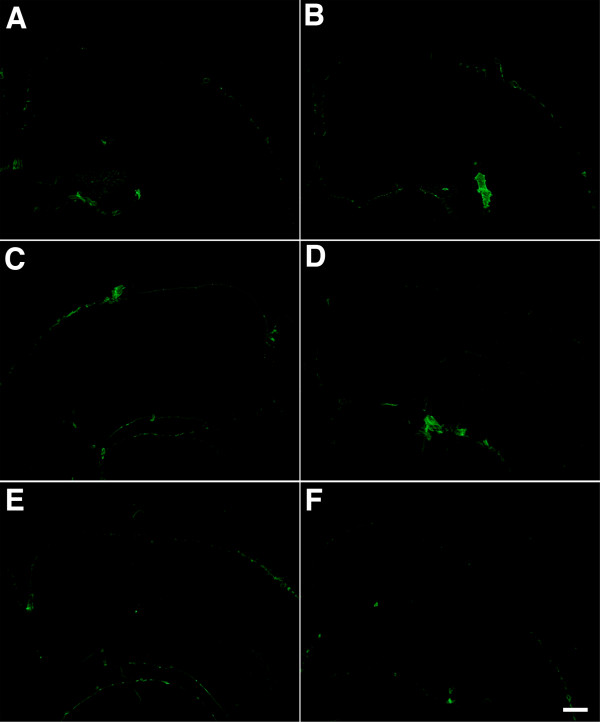
**Lack of collagen IV immunostaining in control brain without pepsin treatment.** Collagen IV immunostaining on a set of serial sections from a control brain that are parallel to those illustrated for the blast-exposed animal in Figure 7. Note the lack of the collagen IV immunostaining in the brain. Scale bar: 750 μm.

Figure 6 shows different magnifications of the cortical region with the lesion illustrated in Figure 5C immunostained for collagen IV without pepsin treatment. A focal lesion is visible in the neocortex of the blast-exposed animal (asterisks in panels B and D). Collagen IV immunostaining is visible not only at the margins of the lesion but extends on both sides of the lesion despite the lack of pepsin pretreatment (panel A). Figure 7 shows collagen IV immunostaining on serial sections taken around the lesion illustrated in Figures 5 and 6. The collagen IV immunostaining in the blast-exposed animal (Figure 7) has significant lateral and rostro-caudal spread around the focal lesion suggesting that there is an extensive area of vascular pathology where the accessibility of the collagen IV antigen to immunostaining is altered. In Figure 8, collagen IV immunostaining is shown on a set of serial sections from a control brain that are parallel to those illustrated for the blast-exposed animal in Figure 7. No vascular staining with collagen IV is apparent in the control brain.

### Ultrastructural microvascular pathology following acute blast exposure

To determine whether ultrastructural changes in the microvasculature are present after blast injury we examined sections of the frontal cortex by EM. Two blast-exposed animals that received a single 74.5 kPa blast exposure and two that received three 74.5 kPa exposures (all sacrificed 24 hours after the last exposure) were studied. One control animal for each exposure condition was processed in parallel.

**Figure 9:**
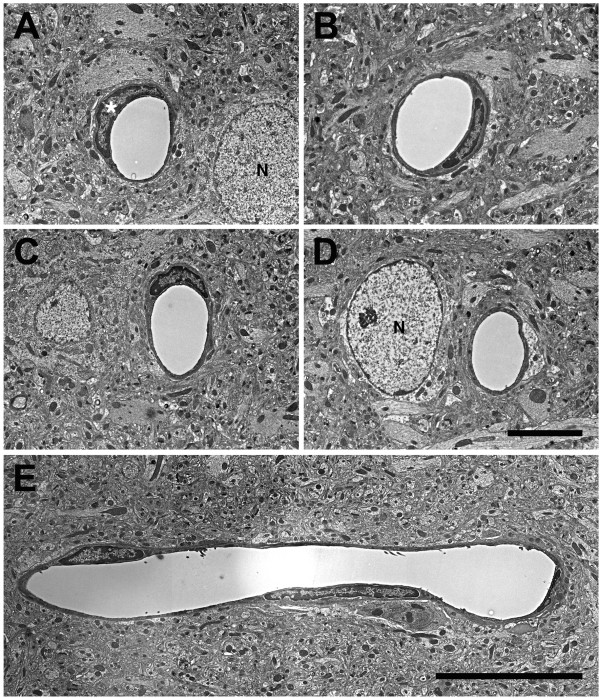
**Normal microvasculature in non-blast exposed adult rat brain.** Examples of normal cerebral microvessels from control rats not exposed to blast overpressure injury are shown cut in cross section **(A-D)** or longitudinally **(E)**. Note the circular lumens, intact endothelial cells and smooth vascular walls. An endothelial cell nucleus is indicated by an asterisk in panel **A**. The nuclei of neurons (N) are labeled in panels **A** and **D**. Scale bars: 6 μm **A**-**D**; 15 μm **E**.

**Figure 10:**
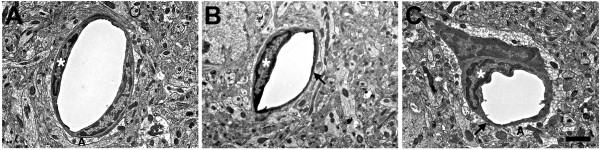
**Luminal alterations in the microvasculature following blast exposure.** Transverse section of a control microvessel **(A)** with its general ultrastructural morphology. Also shown are microvessels from animals that received either one **(B)** or three **(C)** 74.5 kPa blast exposures and were sacrificed 24 hours later. Note the circular lumen of the normal vessel **(A)**. By contrast the luminal circularity has been lost in the microvessels shown in **B** and **C**. Endothelial cell nuclei are labeled with asterisks. Astrocytic processes (A) are indicated in panels **A** and **C**. In panels **B** and **C**, the endothelial cell nuclei are intact although the microvessel walls (arrows in panels **B** and **C**) are abnormally electron dense. The surrounding neuropil appears otherwise normal. Scale bar: 2 μm.

**Figure 11:**
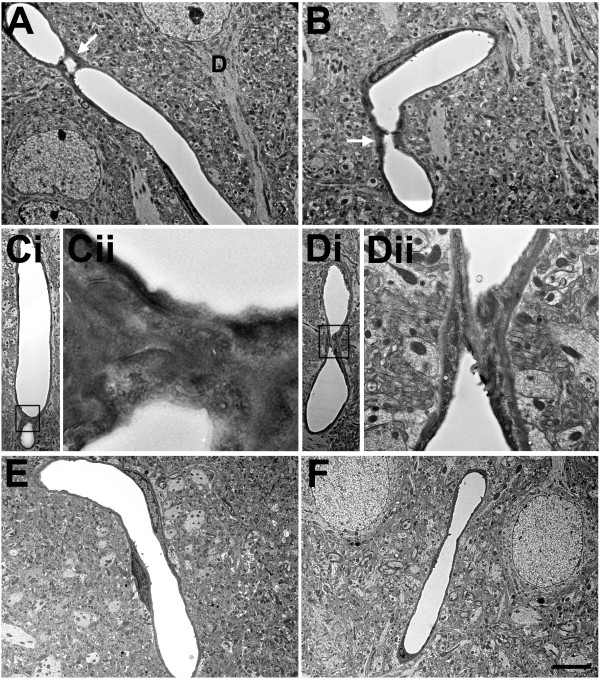
**Microvascular strictures in the blast-exposed brain.** Longitudinal sections of microvessels from animals that were exposed to either one **(A, B)** or three **(C, D)** 74.5 kPa blasts and were sacrificed 24 hours later. Strictures where there is narrowing of the vascular lumen are indicated by arrows. The dendrite (D) of a nearby neuron is indicated in panel **A**. Panel **Ci** shows a region exhibiting a microvascular stricture (box in **Ci**). A higher power image shows that the lumen of this microvessel has been occluded by amorphous material and opposing endothelial cell walls appear to have fused **(Cii)**. Panel **D** shows complete luminal occlusion by amorphous material. The boxed region in panel **Di** is illustrated at higher power in panel **Dii**. Note that despite the destruction of the microvessel architecture at the site of the strictures, the surrounding neuropil appears normal. Panels **E** and **F** illustrate longitudinally cut microvessels from non-blast exposed control brains. Scale bar 1 μm **A**-**B**; 1.2 μm **Ci**; 0.2 μm **Cii**; 2.5 μm **Di**; 0.5 μm **Dii**; 3.5 μm **E**-**F**.

**Figure 12:**
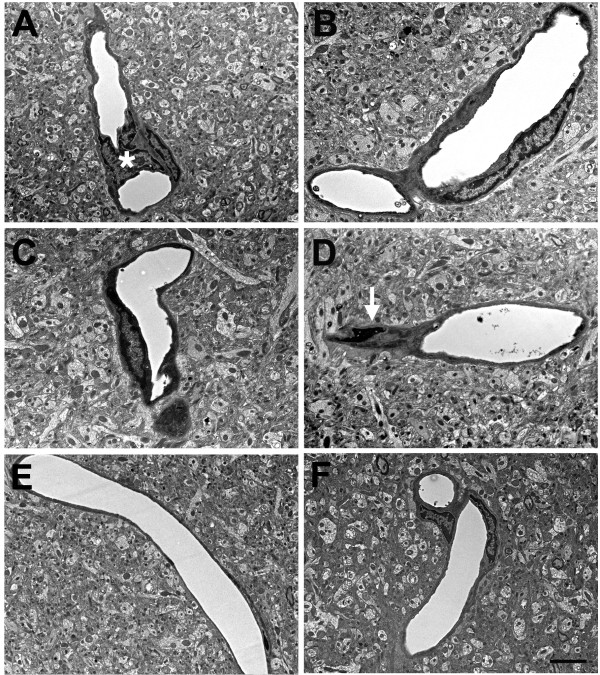
**Blast-induced degenerative changes in cerebral microvessels.** In panels **A**-**D** cerebral microvessels are shown from an animal that received a single 74.5 kPa blast exposure and was sacrificed 24 hours later. Panels **E** and **F** illustrate longitudinally cut cerebral microvessels from non-blast exposed controls. All the microvessels in panels **A**-**D** have lost their luminal circularity and the microvessel walls are irregular. In panel **A**, a dysmorphic endothelial cell nucleus (asterisk) is seen in the lumen of the vessel. In panel **D**, the nucleus of a perivascular cell (arrow) with degenerative changes is indicated. Despite the destruction of the microvessel the surrounding neuropil appears intact. Scale bar: 1 μm **A**-**D**; 3.5 μm **E**-**F**.

**Figure 13:**
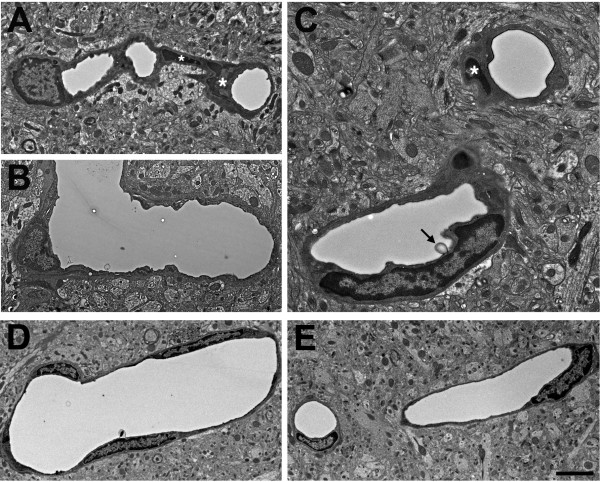
**Advanced degenerative changes in blast-exposed microvessels.** Panels **A**-**C** show cerebral microvessels from an animal that received three 74.5 kPa blast exposures and was sacrificed 24 hours later. Longitudinally cut microvessels from non-blast exposed control brains are shown in panels **E** and **F**. The lumens of the blast-exposed microvessels are irregular. In addition, examples of degenerative changes in perivascular cells are indicated by asterisks in panels **A** and **C**. In panel **C**, an abnormal bulge from the vessel wall is visible in the lumen (arrow). Despite the extensive degenerative changes in the microvessel architecture the surrounding neuropil appears normal. Scale bar: 3.5 μm **A**-**B** and **D**-**E**; 1 μm **C**.

**Figure 14:**
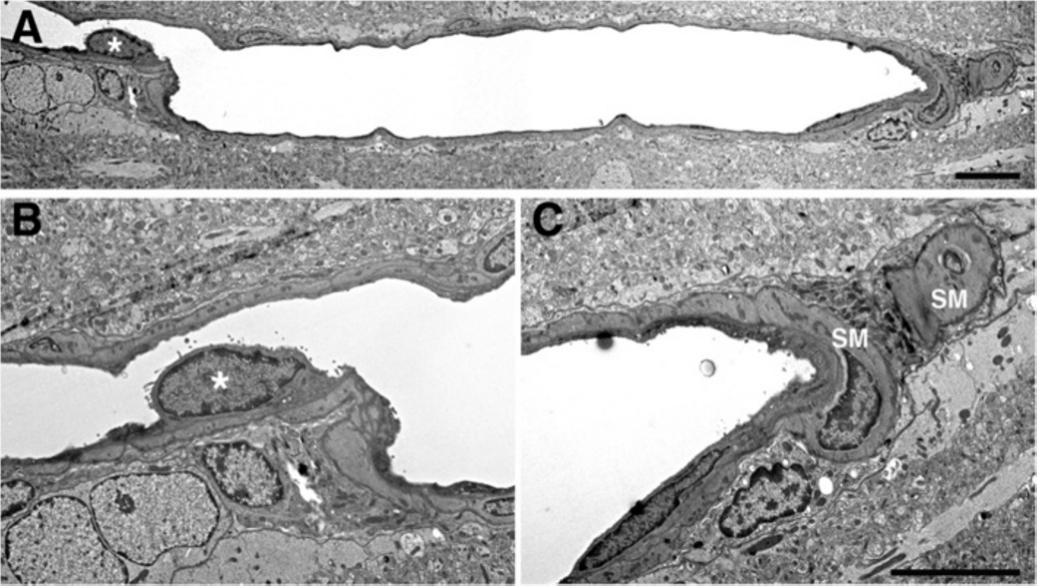
**Blast-exposed microvessel with degenerative changes.** A longitudinal section of a microvessel is shown from an animal that received a single 74.5 kPa blast exposure and was harvested 24 hours later. An endothelial cell nucleus that has been displaced into the vascular lumen is indicated by an asterisk in panels **A** and **B**. At the opposite end of the microvessel the smooth muscle (SM) layers are disrupted. The microvessel lumen is also irregular. Scale bars: 1.5 μm **A**; 3 μm **B**, **C**.

**Figure 15:**
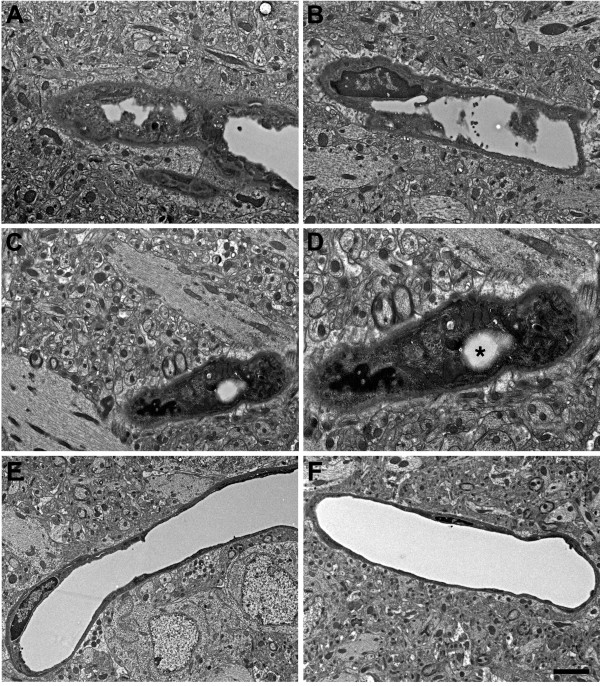
**Microvascular occlusion and degeneration.** Panels **A**-**D** show cerebral microvessels of animals that received 3 × 74.5 kPa blast exposures and were perfused 24 hours later. Note the luminal occlusions by the accumulation of heterogeneous amorphous materials **(A-D)**. Panels **C** and **D** show different magnifications of a remnant of a microvessel with extensive degenerative changes. An apparent lumen indicated by an asterisk in panel **D** is the only architectural feature suggestive of a vascular structure. Endothelial and perivascular cell architecture is otherwise unrecognizable. Despite the extensive degenerative changes in the microvessels the surrounding neuropil appears normal. Panels **E** and **F** illustrate longitudinally cut microvessels from non-blast exposed control brains. Scale bar: 0.9 μm **A**-**C** and **E**-**F**; 0.5 μm **D**.

**Figure 16:**
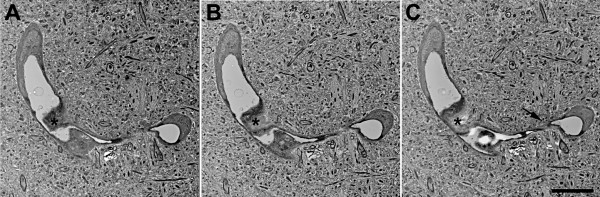
**Chronic microvascular pathology following blast exposure.** Electron micrographs **(A, B, C)** taken from serial sections of the same cortical microvessel. Sections are taken from the frontal cortex of a rat that received three 74.5 kPa blast exposures and was sacrificed 6 months after the last exposure. Note the amorphous material in the lumen creating a near complete occlusion (asterisk). The vessel also becomes narrowed (arrow in panel **C**). The neuropil surrounding the vessel appears normal. Scale bar: 5 μm.

**Figure 17:**
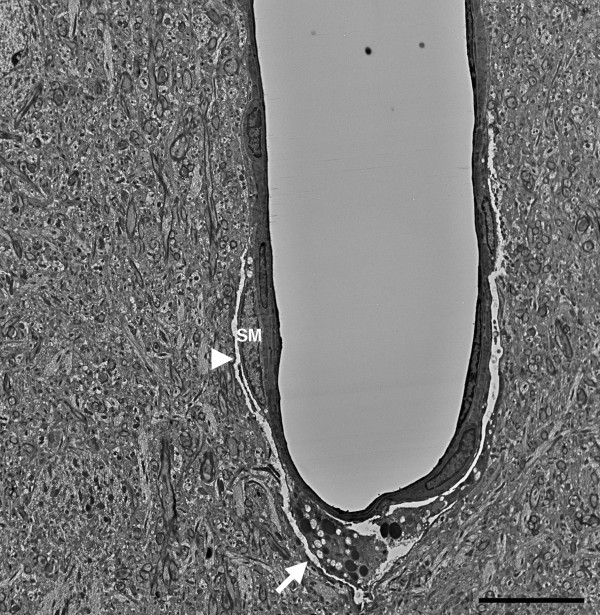
**Chronic pathology in a penetrating cortical vessel following blast exposure.** Transverse section of a cortical penetrating vessel from the frontal cortex of a rat that received three 74.5 kPa blast exposures and was sacrificed 6 months after the last exposure. Note the disruption of the tunica media at the site of the arrow and the presence of a vacuolated region (arrowhead) that extends into the adventitia. A smooth muscle (SM) cell is indicated. The surrounding neuropil appears normal. Scale bar: 10 μm.

Examples of microvessels from control animals are shown in Figure 9. Microvessels in the controls exhibited classic neurovascular ultrastructure with circular lumens, intact endothelial cells and smooth capillary walls. In contrast, in all the blast-exposed rats many microvessels showed varying degrees of pathology (Figures 10, 11, 12, 13, 14, 15, 16 and 17). In the mildest form of the pathology, luminal circularity was lost and the lumens were irregular although the endothelial cell nucleus and the remainder of the microvessel structure were essentially intact. Examples of such vessels are shown in Figure 10. Compared to control (panel A), the luminal circularity is lost in the microvessel in panel B and the lumen of the vessel in panel C is irregular but the microvessels appear otherwise intact except that the vessel walls appear abnormally electron-dense (arrows in Panels B and C).

More severely affected vessels frequently exhibited abnormal strictures where the vascular lumen was narrowed and amorphous material was often present in the lumen at the site of the stricture (Figure 11). Figure 12 shows degenerative changes in microvessel structure. The four microvessels from the blast-exposed animal (panels A-D) have irregular walls in addition to loss of luminal regularity. A dysmorphic endothelial cell nucleus is seen in the lumen of the vessel in Figure 12A. In Figure 12D degenerative changes are seen within the nucleus of a perivascular cell. The nuclear chromatin of this cell appears amorphous and the identity of the cell, which is probably a pericyte, is difficult to recognize. Of note, in all panels, despite the destruction of the microvessel architecture, the surrounding neuropil appears intact.

Figures 13 and 14 show more advanced degenerative changes that extend to perivascular cells. In Figure 13 the blast-exposed microvessel lumens (panels A-C) are irregular with apparent degenerative changes in perivascular cells, which are dysmorphic and display an amorphous chromatin structure. An irregular bulge from the vessel wall is visible in the lumen of one of the microvessels in Figure 13C. In the microvessel illustrated in Figure 14, an endothelial cell nucleus has been displaced into the vascular lumen while at the opposite end of this microvessel the smooth muscle (SM) layer is disrupted. Figure 15 shows additional examples of vascular degeneration associated with luminal accumulation of heterogeneous amorphous material (Figure 15A,B) and extensive degenerative changes in the remnant of a microvessel (Figure 15C,D). In the microvessel shown in Figures 15C,D there is almost complete luminal occlusion where what appears to be a minor luminal opening is the only architectural feature suggestive of a vascular structure while the rest of the vascular and perivascular cell architecture is unrecognizable. Again, despite the extensive degenerative changes in the microvessels in Figures 13, 14 and 15, the surrounding neuropil in all panels appears normal.

### Ultrastructural chronic microvascular pathology following blast exposure

To determine whether chronic microvascular pathology could be found following blast exposure, we examined sections from the frontal cortex of a rat that received three 74.5 kPa blast exposures and was sacrificed 6 months after the last exposure. A microvascular pathology with features similar to that following acute exposure was also observed. One such example is shown in Figure 16 in which amorphous material in the lumen of a microvessel creates a near complete occlusion. In other parts of this vessel the lumen becomes narrow. Chronic ultrastructural changes were also found in penetrating cortical vessels. Figure 17 shows an example of a penetrating cortical vessel with degenerative changes in the tunica media and adventitia. As was found in the setting of acute exposure, despite chronic microvascular degenerative changes the surrounding neuropil appears normal.

## Discussion

In a previous study of rats exposed to single or multiple blast exposures (the approximate equivalent of a human mTBI), we reported that hemorrhages in the choroid plexus and lateral ventricles were one of the most common acute injuries. In the present study we have found that 17% of blast-exposed animals also had blood in the lateral ventricles several months after the last blast exposure, likely indicating continued vascular fragility within the choroid plexus. The choroid plexus consists of a highly vascular leaf-like structure lying in the ventricles where the cerebrospinal fluid is produced [21, 22]. Blood flows to the choroidal arteries from the first rostral branches of the circle of Willis after the internal carotid arteries. The capillaries of the choroid plexus are fenestrated, i.e. non-continuous with gaps between the endothelial cells allowing the free-movement of small molecules. The adjacent choroidal epithelial cells form tight junctions preventing most macromolecules from effectively passing into the cerebrospinal fluid from the blood. The presence of blood in the ventricles of blast-exposed animals even after 10 months post-exposure seems best explained by chronic effects of blast on the vasculature of the choroid plexus resulting in the development of vascular fragility and epithelial damage resulting in the continued blood leakage into the ventricle.

We also previously reported a type of shear-related injury that is common following blast exposure and that has not been described in models of nbTBI [12]. The focal lesions associated with these injuries frequently followed the patterns of penetrating cortical vessels suggesting that the cerebral vasculature is more sensitive to blast-induced injury than other elements in the brain parenchyma. At the very immediate margins of these lesions there is disruption of neuronal as well as non-neuronal elements as would be expected around a lesion where sufficient force was exerted to create the focal rips or tears that were observed [12].

We show that cerebrovascular pathology extends beyond the margins of focal lesions into tissue that exhibits no apparent neuronal or glial pathology. Relying on collagen IV immunostaining, it could be seen that alterations of the vascular extracellular matrix extend well beyond the focal lesions in both the lateral and rostro-caudal directions. By EM, abnormalities in the cerebral microvasculature were apparent in areas distant from focal lesions where no abnormalities were present in neighboring neuronal and glial elements except at the very margins of severely affected blood vessels. Furthermore, these abnormalities were present many months after blast exposure suggesting that they reflect chronic changes in the cerebral microvasculature.

Prior studies have reported a variety of pathological effects associated with blast overpressure injuries [3, 23]. Vascular pathology is a well-recognized component of nb-TBI [24] and high-level blast exposure seems particularly prone to inducing hemorrhagic lesions in humans [25, 26]. Intracranial hemorrhage, altered blood/brain permeability or other forms of vascular pathology have been commented on in a number of studies of experimental blast exposure in animals [6, 10, 27–42]. Elevation of serum and CSF biomarkers of vascular related factors have also been described [38, 43–45]. At the physiological level, acute blast exposure has been associated with prominent vasospasm [46] and decreased cerebral blood flow [47].

Although vascular pathology has been described before, it was mostly shown to occur at relatively high blast exposure levels and in the context of wider neuronal and glial pathology that is not typical of the pathology associated with mTBI. Goldstein et al. [6] have described blast-induced changes in the microvasculature by EM. However, the changes occurred in the context of more general neuronal and glial pathology that included elements of a tauopathy. Our study describes vascular pathology in the absence of widespread neuronal and glial pathology in a model of mTBI and strongly suggests that blast overpressure preferentially damages the vasculature.

Why among the cellular elements in the brain the vasculature would be most susceptible remains unclear. In our prior study we argued that cortically based focal lesions likely represent shear-related effects caused by pressure differentials within the perivascular spaces [12]. We drew this conclusion based on the fact that although the shock wave is transmitted through the entire brain, focal lesions typically followed the course of penetrating cortical vessels. These lesions were seldom associated with hemorrhages as would be expected if the transmitted intravascular pressure wave was sufficient to disrupt the integrity of the vascular structural layers. Increased CSF pressure transmitted through the Virchow-Robin compartments could generate local pressure differentials at the interface between the vascular adventitia and the surrounding tissues resulting in the observed shearing effect.

On the other hand, if the transmitted intravascular pressure was sufficient to rupture the vascular tunicae, it might lead to hemorrhages or to the development of chronic vascular structural alterations that may later result in hemorrhages. It is well known that high hemodynamic stress can cause chronic structural abnormalities in the brain vasculature [48–50]. High blood pressure damages arterial intimal endothelial cells [48–51]. This launches a cascade of events leading to structural alterations that include the proliferation of smooth muscle cells with a pro-inflammatory/pro-matrix remodeling phenotype associated with myointimal hyperplasia, the production of pro-inflammatory molecules, infiltration of mononuclear leukocytes and the accumulation of extracellular matrix with loss of vascular elasticity. Aneurysm formation and progression appear to result from endothelial dysfunction and apoptosis of cellular constituents of the vessel wall [49]. Systemic effects mediated through effects on the vasculature have also been suggested to play a role in blast induced brain injury [52].

In the present study, tortuous vessels with pseudoaneurysms were found next to tear injuries in the brains of blast-exposed animals. Tortuous vessels may signal hypertension as arterial wall thickening and hypertension can force arterioles to assume alternate twisted pathways. The arteriolar tortuosity with pseudoaneurysms together with the presence of foamy vacuolated cells in the tunica media may illustrate the development of vascular fragility and may be a prelude to the later formation of aneurysms. Over time, collagen degradation exceeds synthesis and all of these structural changes contribute to degradation of the integrity of the vascular wall leading to aneurysm dilation and potential rupture causing a hemorrhage [53].

However, pressure transmission through perivascular spaces would not seem to explain the wider microvascular pathology observed here. One pathophysiological clue to the effects of blast may come from the abnormal collagen IV and laminin staining of the microvasculature. The vascular basement membrane is a complex structure of extracellular matrix proteins, proteoglycans, and other structural proteins [54]. Despite their presence in the normal mature adult rodent brain, staining for many basement membrane antigens including laminin and collagen IV occurs only inconsistently in standard perfusion-fixed tissues unless a proteolytic antigen retrieval method is used [14]. Yet, vascular immunostaining of collagen IV or laminin occurred in the brains of blast-exposed rats even without pepsin digestion arguing that a reorganization of the vascular extracellular matrix may have occurred resulting in increased accessibility of these antigens. It is possible that the detection of microvascular extracellular matrix components in non-pepsin treated tissue from blast-exposed animals could result from the action of endogenous degrading proteases such as metalloproteases in the blast-exposed brain. In fact, increases in matrix metalloproteinases and vascular endothelial cell growth factor have been observed acutely following blast [27, 44, 55]. Collectively these observations suggest that blast exposure induces the degradation and remodeling of the extracellular matrix. Interestingly, staining of vessels with antigens such as collagen IV without pepsin treatment is also typical of immature vessels [14] and is also seen in vessels in areas of injured adult brain following mechanical trauma [56] as well as in some models of neurodegeneration [13].

Whereas the physiological basis for these differing patterns of staining are incompletely understood, they seem to correlate with the tightness of the gliovascular junctions (reviewed in [14]). Indeed, one major structural difference between immature and mature microvessels in brain is the large perivascular space present in immature microvessels that separate the outer glial basal lamina and the inner vascular one [56, 57]. In fact, the tightness of the gliovascular junctions seems to correlate with the intensity of laminin immunostaining following mechanical injury [56]. Thus, by analogy, altered collagen IV and laminin staining following blast may reflect a loss of the normal tightness of the gliovascular junctions and could be interpreted as evidence of increased vascular remodeling. Interestingly, decreases in expression of the vascular junctional proteins occludin, claudin-5, and ZO-1 have been reported acutely following blast, changes that would suggest a loss of vascular tight junctions [27].

Why vascular remodeling would be continuing many months after blast exposure as suggested by our findings is unknown. Further examination of the molecular changes that occur particularly in the chronic phase will be needed to understand the pathophysiological basis of this injury. Functional studies will also be needed to determine whether blood–brain barrier function is disrupted as well. However, whatever the underlying basis, these studies suggest that vascular pathology may be a central mechanism in the induction of chronic blast-related injury.

## References

[1] Bochicchio GV, Lumpkins K, O'Connor J, Simard M, Schaub S, Conway A, Bochicchio K, Scalea TM (2008). Blast injury in a civilian trauma setting is associated with a delay in diagnosis of traumatic brain injury. Am Surg.

[2] Jones E, Fear NT, Wessely S (2007). Shell shock and mild traumatic brain injury: a historical review. Am J Psychiatry.

[3] Elder GA, Mitsis EM, Ahlers ST, Cristian A (2010). Blast-induced mild traumatic brain injury. Psychiatr Clin North Am.

[4] Hoge CW, McGurk D, Thomas JL, Cox AL, Engel CC, Castro CA (2008). Mild traumatic brain injury in U.S. Soldiers returning from Iraq. N Engl J Med.

[5] DeKosky ST, Ikonomovic MD, Gandy S (2010). Traumatic brain injury: football, warfare, and long-term effects. N Engl J Med.

[6] Goldstein LE, Fisher AM, Tagge CA, Zhang XL, Velisek L, Sullivan JA, Upreti C, Kracht JM, Ericsson M, Wojnarowicz MW, Goletiani CJ, Maglakelidze GM, Casey N, Moncaster JA, Minaeva O, Moir RD, Nowinski CJ, Stern RA, Cantu RC, Geiling J, Blusztajn JK, Wolozin BL, Ikezu T, Stein TD, Budson AE, Kowall NW, Chargin D, Sharon A, Saman S, Hall GF (2012). Chronic traumatic encephalopathy in blast-exposed military veterans and a blast neurotrauma mouse model. Sci Transl Med.

[7] Omalu B, Hammers JL, Bailes J, Hamilton RL, Kamboh MI, Webster G, Fitzsimmons RP (2011). Chronic traumatic encephalopathy in an Iraqi war veteran with posttraumatic stress disorder who committed suicide. Neurosurg Focus.

[8] Cernak I, Noble-Haeusslein LJ (2010). Traumatic brain injury: an overview of pathobiology with emphasis on military populations. J Cereb Blood Flow Metab.

[9] Chavko M, Koller WA, Prusaczyk WK, McCarron RM (2007). Measurement of blast wave by a miniature fiber optic pressure transducer in the rat brain. J Neurosci Methods.

[10] Ahlers ST, Vasserman-Stokes E, Shaughness MC, Hall AA, Shear DA, Chavko M, McCarron RM, Stone JR (2012). Assessment of the effects of acute and repeated exposure to blast overpressure in rodents: toward a greater understanding of blast and the potential ramifications for injury in humans exposed to blast. Front Neurol.

[11] Elder GA, Dorr NP, De Gasperi R, Gama Sosa MA, Shaughness MC, Maudlin-Jeronimo E, Hall AA, McCarron RM, Ahlers ST (2012). Blast exposure induces post-traumatic stress disorder-related traits in a rat model of mild traumatic brain injury. J Neurotrauma.

[12] Gama Sosa MA, De Gasperi R, Paulino AJ, Pricop PE, Shaughness MC, Maudlin-Jeronimo E, Hall AA, Janssen WG, Yuk FJ, Dorr NP, Dickstein DL, McCarron RM, Chavko M, Hof PR, Ahlers ST, Elder GA (2013). Blast overpressure induces shear-related injuries in the brain of rats exposed to a mild traumatic brain injury. Acta Neuropathol Commun.

[13] Gama Sosa MA, Gasperi RD, Rocher AB, Wang AC, Janssen WG, Flores T, Perez GM, Schmeidler J, Dickstein DL, Hof PR, Elder GA (2010). Age-related vascular pathology in transgenic mice expressing presenilin 1-associated familial Alzheimer's disease mutations. Am J Pathol.

[14] Franciosi S, De Gasperi R, Dickstein DL, English DF, Rocher AB, Janssen WG, Christoffel D, Sosa MA, Hof PR, Buxbaum JD, Elder GA (2007). Pepsin pretreatment allows collagen IV immunostaining of blood vessels in adult mouse brain. J Neurosci Methods.

[15] Chaudhry FA, Lehre KP, van Lookeren CM, Ottersen OP, Danbolt NC, Storm-Mathisen J (1995). Glutamate transporters in glial plasma membranes: highly differentiated localizations revealed by quantitative ultrastructural immunocytochemistry. Neuron.

[16] Janssen WG, Vissavajjhala P, Andrews G, Moran T, Hof PR, Morrison JH (2005). Cellular and synaptic distribution of NR2A and NR2B in macaque monkey and rat hippocampus as visualized with subunit-specific monoclonal antibodies. Exp Neurol.

[17] Hjelle OP, Chaudhry FA, Ottersen OP (1994). Antisera to glutathione: characterization and immunocytochemical application to the rat cerebellum. Eur J Neurosci.

[18] van Lookeren CM, Oestreicher AB, van der Krift TP, Gispen WH, Verkleij AJ (1991). Freeze-substitution and Lowicryl HM20 embedding of fixed rat brain: suitability for immunogold ultrastructural localization of neural antigens. J Histochem Cytochem.

[19] Jucker M, Bialobok P, Hagg T, Ingram DK (1992). Laminin immunohistochemistry in brain is dependent on method of tissue fixation. Brain Res.

[20] Mori S, Sternberger NH, Herman MM, Sternberger LA (1992). Variability of laminin immunoreactivity in human autopsy brain. Histochemistry.

[21] Motti ED, Imhof HG, Janzer RC, Marquardt K, Yasargil GM (1986). The capillary bed in the choroid plexus of the lateral ventricles: a study of luminal casts. Scan Electron Microsc.

[22] Redzic ZB, Segal MB (2004). The structure of the choroid plexus and the physiology of the choroid plexus epithelium. Adv Drug Deliv Rev.

[23] Kobeissy F, Mondello S, Tumer N, Toklu HZ, Whidden MA, Kirichenko N, Zhang Z, Prima V, Yassin W, Anagli J, Chandra N, Svetlov S, Wang KK (2013). Assessing neuro-systemic & behavioral components in the pathophysiology of blast-related brain injury. Front Neurol.

[24] Alves JL (2014). Blood–brain barrier and traumatic brain injury. J Neurosci Res.

[25] Cohen H, Biskind G (1946). Pathologic aspects of atmospheric blast injuries in man. Arch Pathol.

[26] Mott F (1916). The effects of high explosives upon the central nervous system. Lancet.

[27] Abdul-Muneer PM, Schuetz H, Wang F, Skotak M, Jones J, Gorantla S, Zimmerman MC, Chandra N, Haorah J (2013). Induction of oxidative and nitrosative damage leads to cerebrovascular inflammation in an animal model of mild traumatic brain injury induced by primary blast. Free Radic Biol Med.

[28] Valiyaveettil M, Alamneh Y, Wang Y, Arun P, Oguntayo S, Wei Y, Long JB, Nambiar MP (2013). Contribution of systemic factors in the pathophysiology of repeated blast-induced neurotrauma. Neurosci Lett.

[29] Saljo A, Arrhen F, Bolouri H, Mayorga M, Hamberger A (2008). Neuropathology and pressure in the pig brain resulting from low-impulse noise exposure. J Neurotrauma.

[30] Pun PB, Kan EM, Salim A, Li Z, Ng KC, Moochhala SM, Ling EA, Tan MH, Lu J (2011). Low level primary blast injury in rodent brain. Front Neurol.

[31] Garman RH, Jenkins LW, Switzer RC, Bauman RA, Tong LC, Swauger PV, Parks SA, Ritzel DV, Dixon CE, Clark RS, Bayir H, Kagan V, Jackson EK, Kochanek PM (2011). Blast exposure in rats with body shielding is characterized primarily by diffuse axonal injury. J Neurotrauma.

[32] Rubovitch V, Ten-Bosch M, Zohar O, Harrison CR, Tempel-Brami C, Stein E, Hoffer BJ, Balaban CD, Schreiber S, Chiu WT, Pick CG (2011). A mouse model of blast-induced mild traumatic brain injury. Exp Neurol.

[33] Ahmed FA, Kamnaksh A, Kovesdi E, Long JB, Agoston DV (2013). Long-term consequences of single and multiple mild blast exposure on select physiological parameters and blood-based biomarkers. Electrophoresis.

[34] Reneer DV, Hisel RD, Hoffman JM, Kryscio RJ, Lusk BT, Geddes JW (2011). A multi-mode shock tube for investigation of blast-induced traumatic brain injury. J Neurotrauma.

[35] Dalle Lucca JJ, Chavko M, Dubick MA, Adeeb S, Falabella MJ, Slack JL, McCarron R, Li Y (2012). Blast-induced moderate neurotrauma (BINT) elicits early complement activation and tumor necrosis factor alpha (TNFalpha) release in a rat brain. J Neurol Sci.

[36] Kuehn R, Simard PF, Driscoll I, Keledjian K, Ivanova S, Tosun C, Williams A, Bochicchio G, Gerzanich V, Simard JM (2011). Rodent model of direct cranial blast injury. J Neurotrauma.

[37] Cheng J, Gu J, Ma Y, Yang T, Kuang Y, Li B, Kang J (2010). Development of a rat model for studying blast-induced traumatic brain injury. J Neurol Sci.

[38] Ahmed F, Gyorgy A, Kamnaksh A, Ling G, Tong L, Parks S, Agoston D (2012). Time-dependent changes of protein biomarker levels in the cerebrospinal fluid after blast traumatic brain injury. Electrophoresis.

[39] Rafaels KA, Bass CR, Panzer MB, Salzar RS, Woods WA, Feldman SH, Walilko T, Kent RW, Capehart BP, Foster JB, Derkunt B, Toman A (2012). Brain injury risk from primary blast. J Trauma Acute Care Surg.

[40] Readnower RD, Chavko M, Adeeb S, Conroy MD, Pauly JR, McCarron RM, Sullivan PG (2010). Increase in blood–brain barrier permeability, oxidative stress, and activated microglia in a rat model of blast-induced traumatic brain injury. J Neurosci Res.

[41] Skotak M, Wang F, Alai A, Holmberg A, Harris S, Switzer RC, Chandra N (2013). Rat injury model under controlled field-relevant primary blast conditions: acute response to a wide range of peak overpressures. J Neurotrauma.

[42] Turner RC, Naser ZJ, Logsdon AF, DiPasquale KH, Jackson GJ, Robson MJ, Gettens RT, Matsumoto RR, Huber JD, Rosen CL (2013). Modeling clinically relevant blast parameters based on scaling principles produces functional & histological deficits in rats. Exp Neurol.

[43] Svetlov SI, Prima V, Glushakova O, Svetlov A, Kirk DR, Gutierrez H, Serebruany VL, Curley KC, Wang KK, Hayes RL (2012). Neuro-glial and systemic mechanisms of pathological responses in rat models of primary blast overpressure compared to "composite" blast. Front Neurol.

[44] Kovesdi E, Gyorgy AB, Kwon SK, Wingo DL, Kamnaksh A, Long JB, Kasper CE, Agoston DV (2011). The effect of enriched environment on the outcome of traumatic brain injury; a behavioral, proteomics, and histological study. Front Neurosci.

[45] Kovesdi E, Kamnaksh A, Wingo D, Ahmed F, Grunberg NE, Long JB, Kasper CE, Agoston DV (2012). Acute minocycline treatment mitigates the symptoms of mild blast-induced traumatic brain injury. Front Neurol.

[46] Bauman RA, Ling G, Tong L, Januszkiewicz A, Agoston D, Delanerolle N, Kim Y, Ritzel D, Bell R, Ecklund J, Armonda R, Bandak F, Parks S (2009). An introductory characterization of a combat-casualty-care relevant swine model of closed head injury resulting from exposure to explosive blast. J Neurotrauma.

[47] Bir C, Vandevord P, Shen Y, Raza W, Haacke EM (2012). Effects of variable blast pressures on blood flow and oxygen saturation in rat brain as evidenced using MRI. Magn Reson Imaging.

[48] Hashimoto N, Handa H, Nagata I, Hazama F (1980). Experimentally induced cerebral aneurysms in rats: Part V. Relation of hemodynamics in the circle of Willis to formation of aneurysms. Surg Neurol.

[49] Chalouhi N, Ali MS, Jabbour PM, Tjoumakaris SI, Gonzalez LF, Rosenwasser RH, Koch WJ, Dumont AS (2012). Biology of intracranial aneurysms: role of inflammation. J Cereb Blood Flow Metab.

[50] Aoki T, Nishimura A (2011). The development and the use of experimental animal models to study the underlying mechanisms of CA formation. J Biomed Biotechnol.

[51] Hashimoto N, Handa H, Hazama F (1978). Experimentally induced cerebral aneurysms in rats. Surg Neurol.

[52] Cernak I (2010). The importance of systemic response in the pathobiology of blast-induced neurotrauma. Front Neurol.

[53] Abdul-Hussien H, Soekhoe RG, Weber E, von der Thusen JH, Kleemann R, Mulder A, van Bockel JH, Hanemaaijer R, Lindeman JH (2007). Collagen degradation in the abdominal aneurysm: a conspiracy of matrix metalloproteinase and cysteine collagenases. Am J Pathol.

[54] Davis GE, Senger DR (2005). Endothelial extracellular matrix: biosynthesis, remodeling, and functions during vascular morphogenesis and neovessel stabilization. Circ Res.

[55] Prima V, Serebruany VL, Svetlov A, Hayes RL, Svetlov SI (2013). Impact of moderate blast exposures on thrombin biomarkers assessed by calibrated automated thrombography in rats. J Neurotrauma.

[56] Szabo A, Kalman M (2004). Disappearance of the post-lesional laminin immunopositivity of brain vessels is parallel with the formation of gliovascular junctions and common basal lamina. A double-labelling immunohistochemical study. Neuropathol Appl Neurobiol.

[57] Caley DW, Maxwell DS (1970). Development of the blood vessels and extracellular spaces during postnatal maturation of rat cerebral cortex. J Comp Neurol.

